# Supplementary figures and images for: Post-vaccination SARS-CoV-2 IgG spike antibody responses among clinical and non-clinical healthcare workers at a tertiary facility in Kenya
Source: PLoS One. 2024 Apr 4;19(4):e0299302. doi: 10.1371/journal.pone.0299302 (PMC10994319; doi:10.1371/journal.pone.0299302)

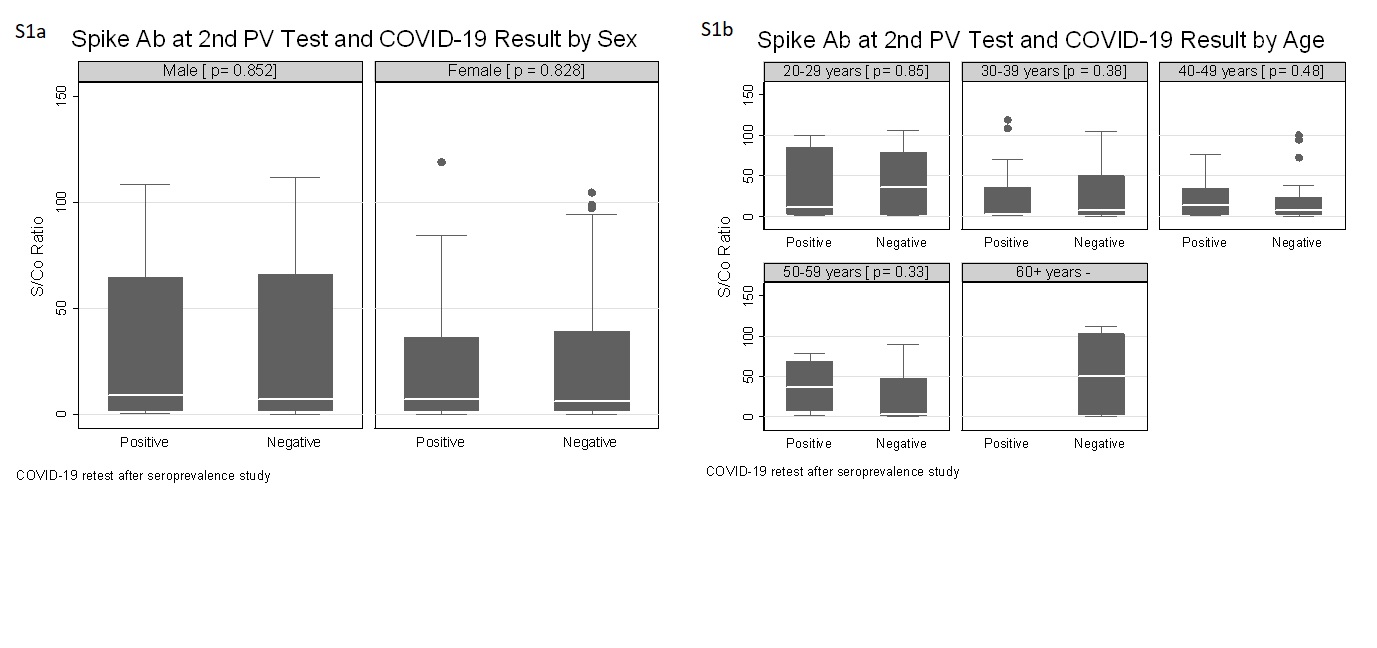

Supplement: S1 Fig — Stratified by sex (S1a) and age category (S1b) [age n = 968]. (JPG) [file pone.0299302.s001.jpg]

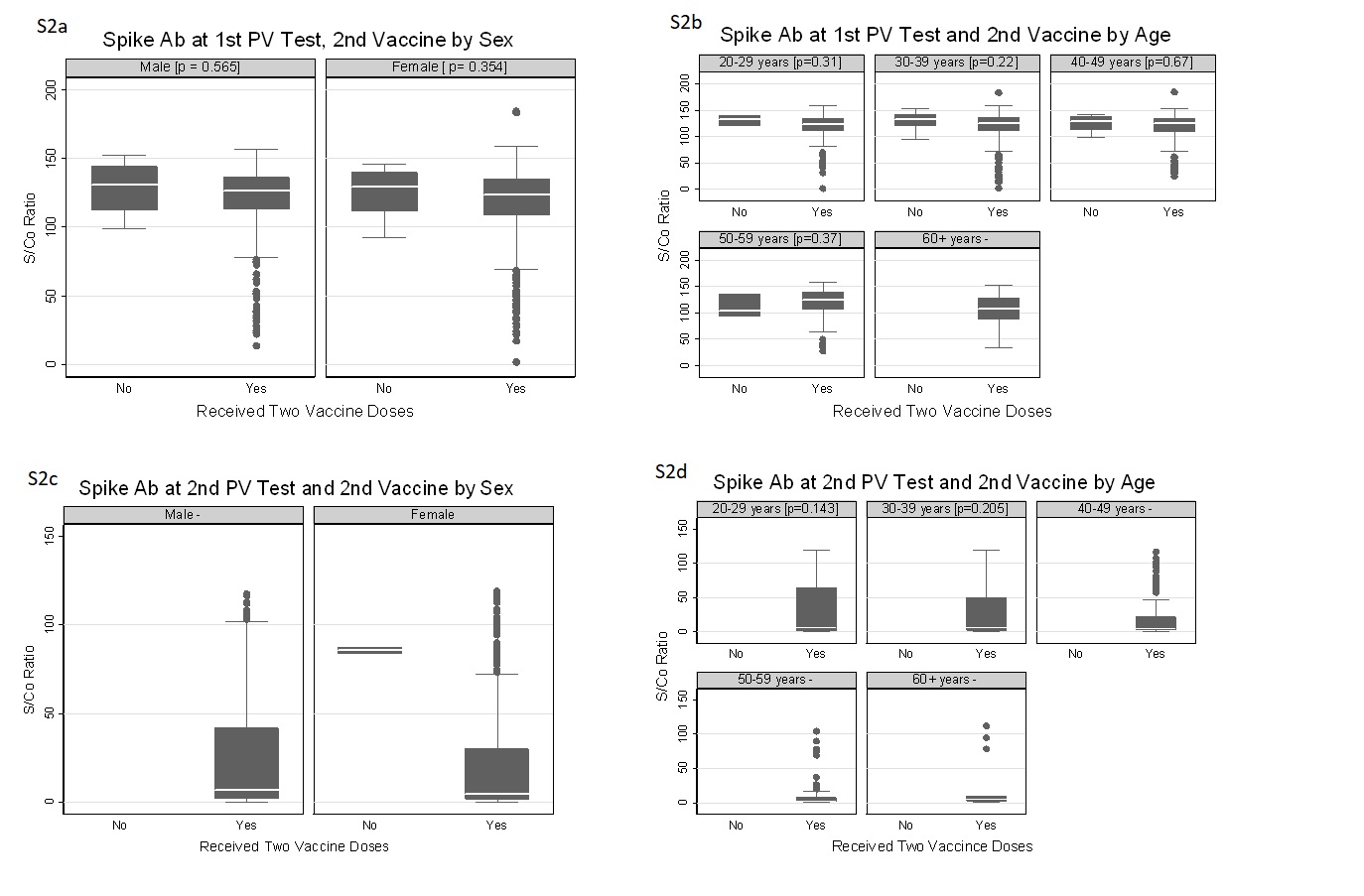

Supplement: S2 Fig — Stratified by sex (S2a and S2c) and age category (S2b and S2d) [age n = 968]. (JPG) [file pone.0299302.s002.jpg]

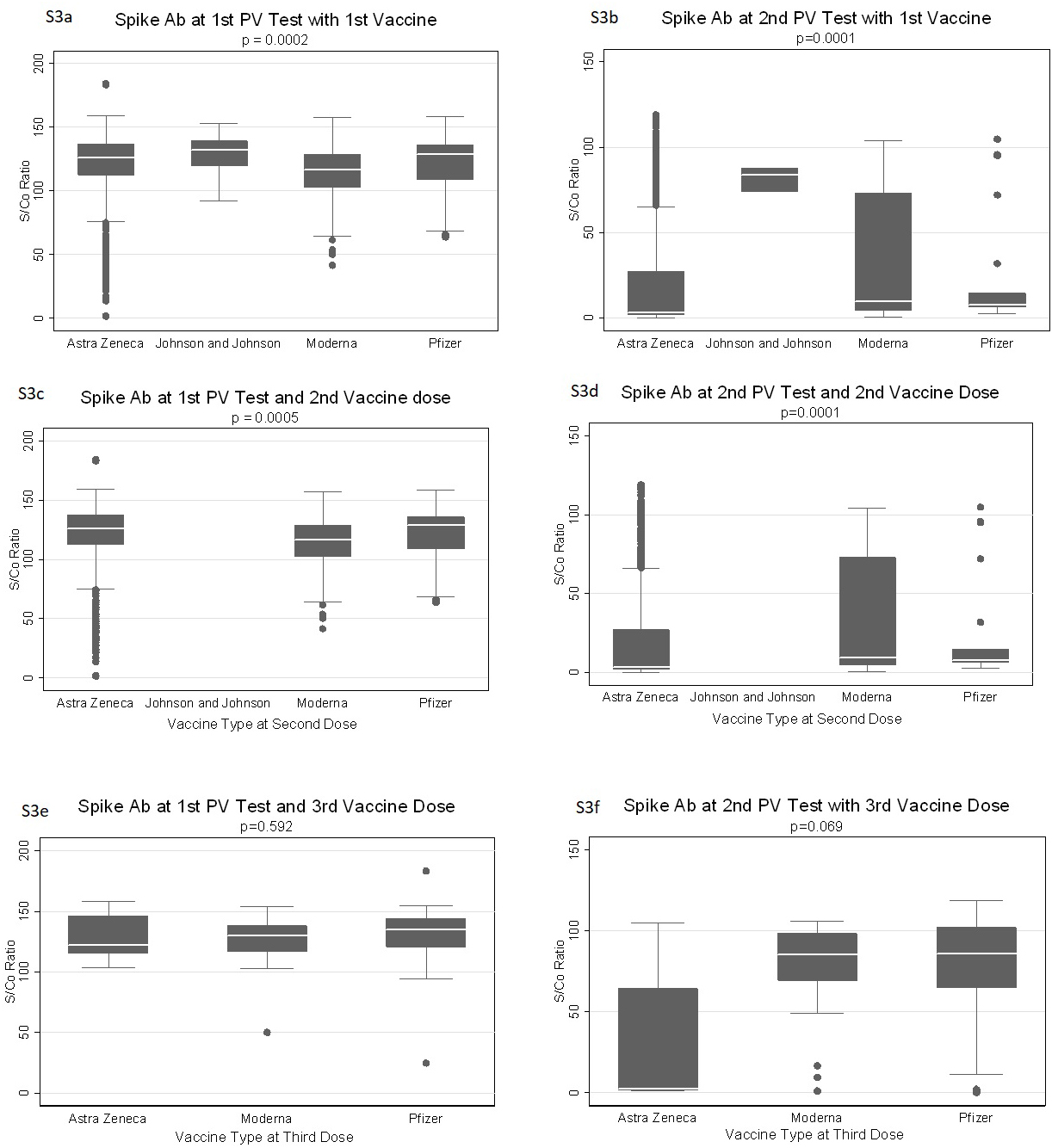

Supplement: S3 Fig — Dose 1 (S3a, S3b), Dose 2 (S3c, S3d) and Dose 3 (S3e, S3f). (JPG) [file pone.0299302.s003.jpg]
