# Supplementary material for: Post-vaccination SARS-CoV-2 IgG spike antibody responses among clinical and non-clinical healthcare workers at a tertiary facility in Kenya
Source: PLoS One. 2024 Apr 4;19(4):e0299302. doi: 10.1371/journal.pone.0299302 (PMC10994319; doi:10.1371/journal.pone.0299302)
Supplement: Attachment — Submitted filename: Reponse to Reviewers.docx [file pone.0299302.s004.docx]

Editor-in-Chief,

PLOS ONE

January 25^th^, 2024

Dear Dr. Chenette,

**RE: SUBMISSION OF REVISED MANUSCRIPT**

We resubmit our manuscript titled: *Post-Vaccination SARS-CoV-2 IgG Spike Antibody Responses among Clinical and Non-Clinical Healthcare Workers at a Tertiary Facility in Kenya*. We thank the reviewers a for their comments after reviewing our manuscript. We submit the revised manuscript for publication; together with the responses to the editor and reviewers, highlighted in blue.

Editor’s comments:

1. Ensure that the manuscript meets PLOS ONE’s style requirements.

*Yes, the manuscript has been edited according to the guidelines.*

2. Thoroughly copyedit the manuscript for language, spelling, and grammar.

*With assistance from a librarian, Mr. Augustine Gitonga, the authors copy-edited the manuscript for grammar, spelling, and flow.*

3. Data Availability Statement.

*Relevant data are within the manuscript and its Supporting Information file.*

4. Review reference list.

*Reference list reviewed.*

Reviewers Comments:

Reviewer #1: *Nice and educative work. It will be nice to also include the benefits of Random Binding Domain/N terminal domain (RBD-NTD vaccines as promising booster vaccines against different variants of SARS COV 2.*

Thank you.
True. RBD-based vaccines present certain benefits against some emerging strains of SARS-CoV-2, therefore promising for vaccine boosting. Our study however was not designed to evaluate the efficacy of boosting mechanisms for the vaccines used for the booster dose.

Reviewer #2:

Thank you for your comments.

a. *The authors did well to mention the limitations of the study. However, in view of these limitations, what are their recommendations for future research in this area?*

This has been addressed – Clean copy, page 11

b. *Inclusion/Exclusion criteria not clearly explained.*

This has been edited to clarify – Clean copy, page 3

Reviewer #3

a. *In table 1, gender shows values of females. It is better to include the data for the male gender as well and do not leave audience to assume the population are only binary and hence the balance is that of males.*

This has been addressed – Clean copy, page 5

b. *The limitation of the study stated under discussion section should be brought out in a separate section probably after the conclusion of the article.*

Thank you. The authors however feel that this can be retained as part of the discussion.

Reviewer #4

This Research Project aligned with the principles of research and publication ethics. Data for the research are readily available. Though I would have appreciate that findings can find a correlation between the high igG spike in the post vaccination and long term immunity against COVID-19.

*All participants had received at least one vaccine dose before enrolment. The study was not designed to establish anti-spike levels prior to vaccination (with natural infection only), and we are not able to distinguish to correlate antibody levels due to vaccination to long term-immunity against SARS-CoV-2.*

Thank you for your consideration. We look forward to hearing from you.

Sincerely,
